# A Comparative and Comprehensive Characterization of Polyphenols of Selected Fruits from the Rosaceae Family

**DOI:** 10.3390/metabo12030271

**Published:** 2022-03-21

**Authors:** Ahsan Hameed, Ziyao Liu, Hanjing Wu, Biming Zhong, Michal Ciborowski, Hafiz Ansar Rasul Suleria

**Affiliations:** 1 Clinical Research Center, Medical University of Bialystok, Jana Kilińskiego Street 1, 15-089 Bialystok, Poland; ahsanhameed@outlook.com (A.H.); michal.ciborowski@umb.edu.pl (M.C.); 2 School of Agriculture and Food, Faculty of Veterinary and Agricultural Sciences, The University of Melbourne, Parkville, VIC 3010, Australia; ziyao.liu@uqconnect.edu.au (Z.L.); hanjingw@student.unimelb.edu.au (H.W.); bimingz@student.unimelb.edu.au (B.Z.)

**Keywords:** polyphenols, LC-MS, HPLC-PDA, plums, peaches, pears, antioxidant activity, antioxidant components, phenolic acids, flavonoids, flavan-3-ols

## Abstract

The present research presents a comprehensive characterization of polyphenols from peach, pear, and plum using liquid chromatography coupled with electrospray ionization quadrupole-time-of-flight-mass spectrometry (LC-ESI-QTOF-MS/MS), followed by the determination of their antioxidant potential. Plums showed the highest total phenolic content (TPC; 0.62 mg GAE/g), while peaches showed the highest total flavonoid content (TFC; 0.29 mg QE/g), also corresponding to their high scavenging activities (i.e., DPPH, ABTS, FRAP, and TAC). In all three fruit samples, a total of 51 polyphenolic compounds were tentatively identified and were mainly characterized from hydroxybenzoic acids, hydroxycinnamic acids, hydroxyphenylpentanoic acids, flavanols, flavonols, and isoflavonoids subclasses. Twenty targeted phenolic compounds were quantified using high-performance liquid chromatography with photodiode array detection (HPLC-PDA). The plum cultivar showed the highest content of phenolic acids (chlorogenic acid, 11.86 mg/100 g), whereas peach samples showed the highest concentration of flavonoids (catechin, 7.31 mg/100 g), as compared to pear. Based on these findings, the present research contributes and complements the current characterization data of these fruits presented in the literature, as well as ensures and encourages the utilization of these fruits in different food, feed, and nutraceutical industries.

## 1. Introduction

Peaches, pears, and plums—members of the Rosaceae family—are widely consumed summer fruits with an enjoyable taste, flavor, and positive health effects. These fruits are commonly consumed as edible fruits and have a relatively high demand in the market [1,2,3,4,5]. These fruits are rich sources of polyphenols, carotenoids, vitamins (A, E, C, and folate), and dietary fiber, which are considered vital constituents responsible for their positive health properties [6,7,8,9]. Polyphenols are one of the main groups of phytochemicals and are present in a diverse range of plants including fruits, vegetables, medicinal plants, among others [10]. Previously, many studies have characterized the polyphenolic composition of peaches, pears, and plums and reported numerous phenolic and flavonoid classes [9,10,11,12,13]. These studies have found peaches to be rich in hydroxycinnamates, flavan-3-ols, flavonols, glucosides, rutin, quercetin, and their derivatives [9,11]. Similarly, pears are considered rich in hydroxycinnamic acids, triterpenoids, and arbutin and their concentration has also been reported in literature [14]. Hydroxycinnamic acids, flavanol, and flavonol were mainly present in the flesh, whereas catechins and procyanidins were abundant in the peel [15]. Regarding plums, the most commonly reported polyphenolic compounds are caffeoylquinic acids, cinnamoyl-hexoses, benzoyl-hexoses hydroxycinnamic acids, *p*-coumaroylquinic acids, flavonol glycosides, and procyanidins [12]. Although these fruits were previously screened for polyphenols, however, the comprehensive characterization was limited. The quality and quantity of polyphenols depend on many factors, including the origins, cultivars, seasons, and/or stage of harvesting of the fruit, as well as many environmental factors, etc. [6,8,16,17,18,19,20,21].

In addition to the above factors, there are various other technical reasons related to the sensitivity, specificity, and accuracy of the instruments, methodological development, sample preparation, and/or treatment approaches which limit the analytical characterization of fruits [22,23]. Therefore, new efforts are always needed to characterize these fruits by addressing these limitations. Comprehensive knowledge about polyphenolic composition (total phenolic content, TPC; total flavonoid content, TFC; and total tannin contents, TTC) would help build predictive computational models that require additional features, such as nutrient-derived metabolic factorial variations and phenotypic changes due to nutritional factors.

Therefore, the primary objectives of the proposed research were to investigate and compare the antioxidant properties including 2,2′diphenyl-1-picrylhydrazyl (DPPH), 2,2′-azinobis-3-ethylbenzothiazoline-6-sulfonic acid (ABTS), ferric-reducing antioxidant potential (FRAP), and the total antioxidant capacity (TAC) of these fruits, followed by their untargeted qualitative characterization analysis using liquid chromatography coupled with electrospray ionization quadrupole-time-of-flight-mass spectrometry (LC-ESI-QTOF-MS/MS) and targeted quantification using high-performance liquid chromatography with photodiode array (HPLC-PDA).

## 2. Results and Discussion

### 2.1. Phytochemical Evaluation

#### 2.1.1. Phenolic Estimation (TPC, TFC, and TTC)

Phenolic contents of fruits and vegetables are mostly measured using in vitro spectrophotometric assays [24,25,26]. TPC, TFC, and TTC of the selected fruit extracts were determined and expressed in mg/g of sample. All the fruit extracts showed a significant variation (*p* < 0.05) in TPC, TFC, and TTC, as shown in Table 1. Previously, Gu and Howell et al. [24] also reported the phenolic content in these three fruits (peach, TPC 0.38 mg gallic acid equivalent (GAE)/g, TFC 0.24 mg quercetin acid equivalent (QE)/g; plum, TPC 0.76 mg GAE/g, TFC 0.28 mg QE/g; pear, TPC 0.34 mg GAE/g, TFC 0.25 mg QE/g). Our work had a similar trend where plum possessed the highest TPC with 0.62 mg GAE/g, followed by peach at 0.43 mg GAE/g and pear at 0.29 mg GAE/g.

In this study, the content of flavonoids was in the range of 0.18 to 0.29 mg QE/g (Table 1), demonstrating a considerable difference (*p* < 0.05) among selected fruits. Of note, the TFC values of peaches and plums were not significantly different (*p* < 0.05). Additionally, peaches were found to have high TFC (*p* < 0.05) compared to plums and pears (Table 1). Previously, many studies reported the TFC of different cultivars of peaches, and showed their potential antioxidative activities [9].

Non-hydrolysable and condensed tannins are high-molecular-weight polymers of the flavan-3-ols catechin, epicatechin, and gallocatechin. The TTC was found in the range of 0.01 to 0.03 mg catechin equivalent (CE)/g (Table 1). The highest concentration of TTC was found in pears, followed by plums and peaches. The concentrations of TTC in our 3Ps (pears, plums, and peaches) samples were less than many fruits and vegetables. In addition, most of the previous studies have not reported any TTC in these fruits [7,9,27,28,29,30,31,32,33,34,35]. One of the reasons might be attributed to the different fruit cultivars, extraction methods, type of material (fresh or dry), seasons, varying cultivation, harvesting, and processing conditions, etc.

#### 2.1.2. Antioxidant Potential (DPPH, ABTS, FRAP, and TAC)

Antioxidant potential can be measured through different mechanisms, including metal chelation, single electron transferring, and hydrogen atom exchanging. In this work, 3P’s extracts were measured by four different assays: DPPH, ABTS, FRAP, and TAC. The results of all assays were expressed as mg of ascorbic acid equivalents (AAE)/g of the samples shown in Table 1. Generally, the highest phenolic contents corresponded to the strongest anti-radical potential of the associated fruit extract. Plums performed the highest DPPH activity at 0.53 mg AAE/g, followed by pears and peaches. It is confirmed that the total phenolic content is proportional to the higher DPPH radical neutralization potential [9,27,28,29,31,36].

The ABTS assay inhibits the oxidation of free ABTS radicals more specifically through hydrogen-donating, which indicates the antioxidant capacity of sample extracts based on the absorbance reduction. The ABTS^•+^ reducing capability of the three different fruit samples varied significantly *(p* < 0.05) from 0.12 to 0.47 mg AAE/g. Plums showed the highest ABTS reducing capacity, followed by peaches and pears. Previously, Curi and Schiassi [37] reported a similar trend in ABTS, where peaches were significantly higher than pears.

The FRAP assay is also widely used to evaluate the reducing capacity of antioxidants via reducing Fe^3+^ into Fe^2+^ by electron transfer [38]. The trend of the FRAP results among the three fruit extracts was similar to DPPH, where the highest FRAP value was reported in plums, followed by pears and peaches. Pellegrini and Serafini [39] also found comparable results that the plums exhibited the highest FRAP values, whereas peaches had significantly higher values than pears. However, with the combination of phenolic estimation, the higher DPPH and FRAP activities of pears could suggest that the flavonoids would probably not be primarily responsible for the antioxidant performance of pears, with the antioxidant activity also being attributed to some other phytochemicals, such as vitamin C [40].

Regarding TAC, significant variations were found in these samples, where plums showed high total antioxidant capacity at 0.41 mg AAE/g. Although peaches showed lower DPPH and FRAP values than pears, the TAC of peaches was significantly higher than pears. This might be due to the contribution of other hydrophilic substances to the TAC of peaches [41].

### 2.2. Characterization and Quantification Using LC-ESI-QTOF-MS/MS and HPLC-PDA

#### 2.2.1. Qualitative Characterization Using LC-ESI-QTOF-MS/MS

Phenolic compounds were tentatively identified and characterized in both the negative and positive mode of ionization, while negative mode data was predominant because of better fragmentation. The tentative identification of compounds was carried out through retention time, *m*/*z* MS, and MS/MS spectra determined by QTOF–MS and using the Agilent LC/MS MassHunter Qualitative Software and PCDL with an online database (Supplementary data; Figures S1 and S2). A total of 51 compounds were tentatively identified and characterized, among which 33 compounds were found in pears, 34 were detected in plums, and only 8 compounds were observed in peaches. Phenolic acids and flavonoids were most prevalent among the total identified compounds. The compounds found in the phenolic acids class were derivatives of hydroxybenzoic acids (8), hydroxycinnamic acids (16), and hydroxyphenylpropanoic acids (3). For the flavonoids class, the major detected compounds were derivatives of dihydrochalcones (3), flavanols (7), flavanones (1), flavones (3), isoflavonoids (3), hydroxybenzaldehydes (1), and hydroxycoumarins (1), as detailed in Table 2.

#### 2.2.2. Phenolic Acids and Derivatives

Phenolic acids and their derivatives have been recognized as the most prevalent phytometabolites in fruits. A total of three subclasses of phenolic acids were tentatively identified and characterized in our work, as shown in Table 2. These subclasses were hydroxybenzoic acids, hydroxycinnamic acids, and hydroxyphenylpropanoic acids.

##### Hydroxybenzoic Acids

Hydroxybenzoic acids have been widely detected in different fruits with significant antioxidant potential [25]. They were the second most abundant phytochemicals in this study and were only found in pear and plum extracts. Compound **3**, corresponding to [M–H]^−^ at *m*/*z* 169.0145, was tentatively identified as gallic acid, which is a well-known antioxidant in phytochemistry. This compound showed characteristic fragment ions in the product ion spectra by the consecutive loss of CO_2_, gallate, and galloyl moieties [42,43]. Previously, Yang and Jayaprakasha [44] has also confirmed and identified gallic acid and gallic-acid-based phenolic acids in the ten cultivars of pear [42,43]. Similarly, other gallic-acid-based phenolic acids were also tentatively identified in pears, showing the loss of the galloyl moiety (152 Da) and CO_2_ (44 Da) from precursor ions (i.e., gallic acid 3-*O*-gallate; compound **7**). The product ions of this compound were detected at 169 and 125 *m*/*z*, consistent with data reported previously in the literature [42,45,46]. Compound **1** (*m*/*z* 331.0672) exhibited a product ion at *m*/*z* 169 and *m*/*z* 125, by losing glucoside (162 Da) and the consecutive loss of CO_2_ (44 Da) in negative ion mode, and, as such, was tentatively identified as gallic acid 4-*O*-glucoside. Recently, a study related to the authentication of pear juices and peach purees has cited various glucosides as “biomarkers” for the commercial varietal pear juices and peach purees [47]. However, no exact similarity in the detected compounds was seen, which may be attributed to the two different analytical platforms used in the two studies. Compound **8** was tentatively identified as 2,3-Dihydroxybenzoic acid, with its [M–H]^−^ at *m*/*z* 153.0190 yielding product ions at *m*/*z* 109, with a major loss of carbon dioxide (44 Da) [6,48].

##### Hydroxycinnamic Acids

Numerous studies have reported hydroxycinnamic acids as the most abundant phenolic acids in fruits (i.e., caffeic acid and ferulic acid), significantly contributing to their antioxidant potential [49]. This subclass of compounds is comprised of a larger number of detected compounds than any other subclass in this study. A total of 16 derived compounds of hydroxycinnamic acids were tentatively identified in this work. Compounds **15** (*m*/*z* 337.0934) and **20** (*m*/*z* 367.1025) were quinic acid derivatives, referred to as 3-*p*-coumaroylquinic acid and 3-feruloylquinic acid, respectively. The tentative identity of 3-caffeoylquinic acid (compound **12**) was further confirmed, based on its fragmentation pattern of (*m*/*z* 253, 190, and 144), and (*m*/*z* 298, 288, 192, and 191), respectively. In terms of 3-*p*-coumaroylquinic acid, the product ions at *m*/*z* 265 [M–H−72], *m*/*z* 173 [M–H−164], and *m*/*z* 162 [M–H−175] were due to the loss of 4H_2_O, C_9_H_7_O_3_, and C_7_H_11_O_5_, respectively [50]. The tentative identity of 3-feruloylquinic acid (compound **20**) was confirmed by the fragments at *m*/*z* 298 [M–H−3H_2_O_2_−CH_3_, loss of 69 Da], *m*/*z* 288 [M–H−H_2_O−CH_3_−HCOOH, loss of 79 Da], *m*/*z* 192 [M–H−C_7_H_11_O_5_, loss of 175 Da], and *m*/*z* 191 [M–H−C_10_H_8_O_3_, loss of 176 Da] [50]. Previously, Kolniak-Ostek and Oszmiański [19] have also identified several isomers of caffeoylquinic acid in anatomical pears, based on their retention time and fragmentation pattern in MS^n^. We also tentatively identified the cis-forms of 3-*p*-coumaroylquinic acid and 3-feruloylquinic acid in the flesh and peel of pears. Our findings of compounds with quinic/chlorogenic acid moieties also agreed with Brahem and Renard [51], who detected 3-*p*-coumaroylquinic acid and 3-feruloylquinic acid in the flesh and peel of Tunisian and European pear cultivars. In plums, four caffeoylquinic acids, one feruloylquinic acid isomer, and four peaks for *p*-coumaroylquinic acid have also been cited previously by Jaiswal and Karaköse [12]. As per our knowledge, our work is the first to report the presence of cinnamic acid (compound **10**) in fresh plums based on its molecular ion ([M–H]^−^ at *m*/*z* 147.0444) and its product ions (*m*/*z* 103), showing a typical loss of CO_2_ (44 Da) [52]. Previously, cinnamic acid or its derivatives, have only been reported in the fermented pomace of plums [12,53]. Five compounds with ferulic structures were characterized as ferulic glycosides, with [M–H]^−^ at *m*/*z* 273.0067 (compound **9**), 325.0567 (compound **11**), 193.0492 (compound **18**), 355.1010 (compound **19**), and 369.0826 (compound **22**) tentatively identified as isoferulic acid 3-sulfate, feruloyl tartaric acid, ferulic acid, ferulic acid 4-*O*-glucoside, and ferulic acid 4-*O*-glucuronide, respectively. Similarly, our fragmentation spectra of ferulic acid (compound **18**) also showed product ions with *m*/*z* 178, 149, and 134, indicating the loss of CH_3_, CO_2_, and CH_3_ with CO_2_ from the precursor, respectively, as has also been evidenced by Sasot and Martínez-Huélamo [52]. Compound **11**, with pseudo-molecular ions at *m*/*z* 325.0567 fragmented in *m*/*z* 193 and 149, indicates the presence of ferulic acid and tartaric acid ions [54,55]. The characteristic fragmentation pattern of isoferulic acid 3-sulfate showed product ion spectra at *m*/*z* at 193 and 178, with a loss of the sulfate unit (80 Da) and further loss of (15 Da) CH_3_, which is in line with the findings of Sasot and Martínez-Huélamo [52].

#### 2.2.3. Flavonoids

Flavonoids were the second major group of polyphenols found in the selected extracts. They were divided into eight subclasses, including flavanols, flavones, flavanones, flavonols, dihydrochalcones, dihydroflavonols, anthocyanins, and isoflavonoids. Our LC-MS data analysis found six subclasses of flavonoids such as dihydrochalcones, flavanols, flavanones, flavones, flavonols, and isoflavonoids. We found 25 polyphenolic compounds from these six subclasses in the studied extracts. Unlike the phenolic acids and their derivatives, a greater number of compounds of this class were found in peaches.

##### Dihydroflavonols and Dihydrochalcones

A total of three compounds were observed in this sub-class of flavonoids, based on QTOF-MS analysis followed by MS/MS. Compound **28** was tentatively identified as dihydroquercetin, with its [M–H]^−^ at *m*/*z* 303.0501 and its MS/MS product ions at *m*/*z* 285, 275, and 151. The product ions at *m*/*z* 285 and 275 corresponded to a loss of H_2_O (18 Da) and CO (28 Da), with the 152 Da loss attributed to retro-Diels-Alder (RDA) cleavage [56,57]. Compound **29** showed an [M–H]^−^ at *m*/*z* 451.1258, which produced fragment ions at *m*/*z* 289 [M–H−glucoside] and *m*/*z* 273 (phloretin aglycon), tentatively confirming the identity of this metabolite as 3-hydroxyphloretin 2′-*O*-glucoside [57]. Compound **30**, with RT = 51.681 min and molecular ion [M–H]^−^ at *m*/*z* 435.1307 showing the fragmentation ion at *m*/*z* 273 (phloretin aglycon) with the expected loss of glucoside (162 Da), was tentatively assigned the name phloridzin [57].

##### Flavanols

Flavanols, or flavan-3-ols, are the most common flavonoids, which feature diversity in their chemical structures and biological functions [58]. The types of flavanols found in our samples were monomeric flavanols, consisting of catechin, epicatechin, epigallocatechin, gallate, gallocatechin derivatives, and their polymerized products in the form of dimers and trimers. In this study, collectively, seven flavanols were tentatively identified in all three fruits, including polymerized and derivative compounds. Compounds **31** and **32**, having RT = 15.562 and 19.24 at *m*/*z* 577.1348 and 865.1961, respectively, were tentatively identified as procyanidin dimer B1 and procyanidin trimer C1, with MS/MS fragment ions at *m*/*z* 451 and 739, 713, and 695, respectively. The fragmented ion at *m*/*z* 451 represented the recognized loss of a phloroglucinol (126 Da) fragment, while product ions at *m*/*z* 739, *m*/*z* 713, and *m*/*z* 695, representing the 126 Da loss of heterocyclic ring fission (HRF) reaction, 152 Da loss of RDA, and further loss of H_2_O, tentatively confirmed the identity of these procyanidin polymeric compounds in all three fruits [59]. These identifications were also in line with the studies of Jaiswal and Karaköse [12] and Zhao and Zhang [9], who earlier identified dimeric and trimeric proanthocyanidins in plums and peaches. The presence of procyanidin dimer B1 and procyanidin trimer C1 in pears also agreed with previous studies available in the literature [18,19]. These studies found A- and B-type dimers, trimers, and tetramers, while the recently published work by Amaya-Cruz and Pérez-Ramírez [60] did not find any procyanidin polymerized compounds in prickly pear varieties. Compound **34** at RT = 19.684 min, with a pseudomolecule ion at *m*/*z* 289.0706, was allocated for (+)-Catechin, the most common isomer of catechin, based on the fragmentation pattern that showed the product ion at *m*/*z* 245, *m*/*z* 205, and *m*/*z* 179, correspond to the loss of CO_2_ (44 Da), flavonoid a ring (84 Da), and flavonoid b ring (110 Da). Catechins are the building blocks of proanthocyanidins, a type of condensed tannin. (+)-catechin and (+)-gallocatechin are known to be the most potent antioxidants, with anti-diabetic, anti-cancer, anti-inflammatory, and other health-promoting activities [61]. The parent ion of compound **35**, ((+)-gallocatechin), was detected at *m*/*z* 305.0648, with a fragmentation spectrum and production ion distribution at *m*/*z* 261 and 219, with the respective loss of one CO_2_ and C_3_H_2_O_3_, which is consistent with the values published by Kelebek [62]. Compound **36**, having the parent ion [M–H]^−^ at *m*/*z* 303.0878, was tentatively identified as 3′-*O*-Methylcatechin, a metabolite of (+)-catechin. The product ions of this compound were seen at *m*/*z* 271 [M–H−CH_3_OH, loss of 32 Da] and *m*/*z* 163 [M–H−CH_3_OH−C_6_H_5_O_2_, loss of 140 Da] [63]. This compound is a well-known enzyme inhibitor and anti-ulcer agent, as well as a urinary biomarker of fruit and tea consumption [64]. Compound **37** was characterized as (+)-catechin 3-*O*-gallate, due to its parent ion at *m*/*z* 441.0805 and product ions at *m*/*z* 289 [M–H−C_7_H_5_O_4_], *m*/*z* 169 [M–H−C_7_H_5_O_4_−C_8_H_8_O], and *m*/*z* 125 [M–H−C_7_H_5_O_4_−C_8_H_8_O−CO_2_], as previously reported [65,66].

##### Flavonols

Flavonols were also prevalent flavonoids detected in all three selected fruits in this study. By comparing the flavonoids literature, the most detected flavonols in this study were aglycone derivatives of kaempferol, myricetin, and quercetin. These aglycone derivatives are well-known for their particularly potent anti-diabetic effects. Some studies have cited these aglycone derivatives as eight times stronger than the anti-diabetic drug acarbose [67]. Isorhamnetin (compound **42**, [M–H]^−^ at *m*/*z* 315.0504) was found in plum in negative modes and was identified according to the product ions at *m*/*z* 300 and *m*/*z* 271, corresponding to the loss of CH_3_ and CO_2_ from the precursor [68]. Compound **44** was tentatively identified as myricetin 3-*O*-rhamnoside, at *m*/*z* 463.0847. Its putative identification was further confirmed by the pattern of its fragmentation spectra and neutral loss of rhamnoside (146 Da) [M–H−317] from the product ion [69]. Myricetin 3-*O*-rhamnoside has significant medicinal, nutritional, and health-promoting activities, as it involves repairing iron-induced DNA oxidation, inhibits digestive, lipid, fecal, and colonic bacterial enzyme activities, and acts as an anti-allergenic, anti-cancer, and anti-obesity compound [70]. Of note, myricetin and its derivative compounds, have not been identified in any previous studies of the three fruits, except for the study of Amaya-Cruz and Pérez-Ramírez [60], in which myricetin 3-*O*-rhamnoside was found in prickly pears. Two compounds with the quercetin moiety were tentatively identified—namely, quercetin 3-*O*-glucosyl-xyloside (compound **43**; RT = 34.73, *m*/*z* 595.1290) and quercetin 3-*O*-arabinoside (compound **46**; RT = 45.598, *m*/*z* 433.0780)—in plums and pears. The MS/MS spectra confirmed the identity of quercetin 3-*O*-glucosyl-xyloside, which showed a product ion at *m*/*z* 265 [M–H−glucose−xylose, loss of 330] [50]. Similarly, the MS/MS spectra of quercetin 3-*O*-arabinoside also showed the product ion [M–H]^−^ at *m*/*z* 301 [M–H−arabinoside, loss of 132] [71]. Both of these flavonoid-3-*O*-glycosides (compounds **43** and **46**) have a flavonoid moiety that is *O*-glycosidically linked to the carbohydrate moiety at the C3-position. Most previous studies have found flavonols with quercetin and kaempferol linked to rutinosides, galactosides, and glucosides; however, both flavonoid-3-*O*-glycosides, as mentioned above, have not previously been identified in plums and/or pears.

##### Isoflavonoids

The biogenetic derivation of the 3-phenylchroman skeleton from the 2-phenylchroman skeleton of flavonoids leads to the formation of a particular sub-class of flavonoids called isoflavonoids (or phytoestrogens). Generally, most of the isoflavonoids in nature are present in *β*-D-glycoside form, such as daidzin, genistin, and glycitin. In total, three isoflavonoids were only tentatively identified in plums and pears in this study. This is the first study of this kind to identify isoflavonoids in plums and pears, as no previous studies have identified any isoflavonoids in these fruits [12,17,18,19,31,60]. Compounds **47** and **48** both appeared in plum and were detected in negative mode with tentative identifications as 6″-*O*-acetyldaidzin and violanone according to the [M–H]^−^ at *m*/*z* 457.1125 and *m*/*z* 315.0868, respectively. The identification of 6″-*O*-acetyldaidzin was achieved by the fragment at *m*/*z* 221, corresponding to the loss of C_15_H_8_O_3_ (236 Da) from the precursor. In the MS^2^ spectra, product ions at *m*/*z* 300 [M–H−CH_3_, loss of 15 Da], *m*/*z* 285 [M–H−2CH_3_, loss of 30 Da], and *m*/*z* 135 [M–H−C_10_H_12_O_3_, loss of 180 Da] allowed for the identification of violanone. 6″-*O*-acetyldaidzin (or daidzein 6″-*O*-acetate) is naturally derived from 3-phenylchromen-4-one, due to the replacement of (phenolic) hydrogen at position 7 by a 6-*O*-acetyl-beta-D-glucosyl residue [72]. Compound **49** was tentatively identified as glycitin based on the precursor at [M–H]^−^ at *m*/*z* 445.1150, and further confirmed by the peak at *m*/*z* 285 after the loss of a glucosyl group, as has also been reported by Ren and Wang [73]. Daidzin and glycitin 7–*β*-glucosides are most abundant in the hypocotyl of seeds, whilst the cotyledon contains more genistin isoflavonoids. Glycitin has been reported to be deconjugated to aglycones/glycitin by the action of intestinal *β*-glucosidases, lactase phlorizin hydrolase, and/or glucocerebrosidase [74].

#### 2.2.4. Other Polyphenols

The other detected polyphenols included hydroxybenzaldehydes (1) and hydroxycoumarins (2). 4-Hydroxybenzaldehyde (Compound **50**) was found in both negative and positive modes, and was tentatively identified according to the precursor [M–H]^−^ at *m*/*z* 121.0298. In the MS^2^ experiment of 121.0298, a product ion at *m*/*z* 77 achieved the confirmation of 4-hydroxybenzaldehyde, which exhibited the loss of CO_2_ (44 Da) from the precursor. This compound is involved in dakin oxidation, an organic redox reaction, where it reacts with NAD^+^ and H_2_O to produce 4-hydroxybenzoate, NADH, and two protons [42,52]. Coumarin (Compound **51**), with [M–H]^+^ ion *m*/*z* at 147.0448, was detected in the plums. The identity of coumarin was confirmed with fragments at *m*/*z* 103 and at *m*/*z* 91, which represented the loss of CO_2_ and 2CO, respectively. Coumarins are fused benzene and α-pyrone rings, and almost 1300 coumarins have been identified in nature. Coumarins have anti-inflammatory, anti-viral, anti-thrombotic, and vasodilatory activities.

### 2.3. Quantitative Characterization of 3Ps Extracts Using HPLC-PDA

In this study, a total of 20 phenolic compounds including 10 phenolic acids and 10 flavonoids were selected for quantification purposes in the selected fruits. Among this, 17 phenolic compounds were quantified in peaches, 16 in pears, and 18 in plums using HPLC-PDA. The content of each phenolic compound is expressed as mg per 100 g of fresh sample basis ± standard deviation, and the total phenolic acids and flavonoids were calculated by summing the individual phenolic acids and flavonoids together as shown in Table 3.

For the quantification of phenolic acids, plums contained the most abundant total phenolic acids, followed by peaches and pears. Furthermore, plums also showed the highest concentration of six individual phenolic acids out of ten, including caftaric acid, *p*-hydroxybenzoic acid, ferulic acid, chlorogenic acid, caffeic acid, and coumaric acid. Kim and Chun [75] have also quantified the chlorogenic acid in twelve varieties of fresh plums and found them in the range of 0.9 to 21 mg/100 g, which was consistent with our findings. The primary phenolic acids found in peaches were *p*-hydroxybenzoic acid, sinapic acid, and syringic acid, while three phenolic acids, including protocatechuic acid, caffeic acid, and coumaric acid, were not identified in peaches. Pears were only rich in gallic acids and chlorogenic acid. Sun-Hee and Yim [76] also reported a comparable amount of gallic acid as 7.85 to 9.40 mg/100 g _D.W_ and chlorogenic acid as 5.51 to 16.86 mg/100 g _D.W_.

Regarding the quantification of flavonoids, peaches showed the highest content of flavonoids while plums exhibited the lowest. Individually, the most prominent flavonoids quantified in pears included polydatin, epicatechin, and quercetin-3-*O*-rhamnoside, whereas resveratrol and quercetin were absent, which was consistent with Sun-Hee and Yim [76]. A total of five flavonoids were found in peaches, including catechin, quercetin-3-*O*-galactoside, quercetin-3-*O*-rhamnoside, kaempferol-3-*O*-glucoside, and kaempferol. Previously, Rodríguez-González and Pérez-Ramírez [77] determined the concentration of quercetin in peach juices varying from 2.9 to 5.0 µg/100 g, which was significantly lower than our findings. However, Zhao and Zhang [9] detected a significantly higher content of catechin (60.14 to 1030.03 mg/kg _D.W_) and chlorogenic acid (52.20 to 1264 mg/ kg _D.W_) in various varieties of peaches compared to our results.

Noticeably, although only quercetin-3-*O*-rhamnoside was not detected in plums, the rest of the flavonoids’ content was significantly lower than its concentration of phenolic acids in the other two fruits. In contrast, flavonoids dominated the phenolic constitution in peaches. Moreover, phenolic acids and flavonoids share similar content in pears.

## 3. Materials and Methods

### 3.1. Chemicals and Reagents

Analytical grade solvents and reagents used for extraction purposes were supplied by Sigma-Aldrich (Castle Hill, NSW, Australia). The reference compounds are quercetin, gallic acid, L-ascorbic acid, protocatechuic acid, gallic acid, *p*-hydroxybenzoic acid, caftaric acid, caffeic acid, chlorogenic acid, coumaric acid, syringic acid, epicatechin gallate, quercetin-3-*O*-glucuronide (q-3-*O*-glucuronide), kaempferol-3-*O*-glucoside, quercetin-3-galactoside, quercetin, kaempferol, and catechin. In addition, aluminium chloride hexahydrate, Folin and Ciocalteu’s (FC) phenol, 2,2-diphenyl-1-picrylhydrazyl (DPPH), vanillin, 2,4,6-tripyridyl-s-triazine (TPTZ), ferric (III) chloride anhydrous, 2,2′-azino-bis(3-ethylbenzthiazoline-6-sulphonate) (ABTS), and potassium persulfate were purchased from Sigma-Aldrich (St. Louis, MO, USA). Sodium acetate hydrated (Ajax Finechem, Scoreby, VIC, Australia), sodium carbonate anhydrous (Chem-supply; Gillman, SA, Australia), and sulfuric acid 98% (Sigma-Aldrich, St. Louis, MO, USA) were purchased from RCI Lab scan Limited, (Bangkok, Thailand). The mobile phases and eluents were HPLC grade and purchased from Fisher Chemical Company (San Jose, CA, USA). The mobile phases for both analytical platforms (HPLC and LC-ESI-QTOF-MS/MS) were acetic acid/water solution (2:98 *v*/*v*, mobile phase A) and acetonitrile/water/acetic acid (50:49.5:0.5, *v*/*v*/*v*, mobile phase B) (LiChrosolv, Darmstadt, Germany). Ultrapure (UP) water purified from a Millipore Milli-Q Advantage A10 Water Purification System (Bedford, MA, USA) was used throughout.

### 3.2. Sample Preparation

Fully matured and ripened fruits (peaches—Summer Sweet; pear—Packham’s Triumph; and plum—Queen Rosa) were selected for this study. The freshly harvested fruits were acquired from local farmers and were grown in Victoria, Australia. The fresh fruits (2–3 kg of each) were blended (1.5 L blender, Russell Hobbs Classic, model DZ-1613, Melbourne, VIC, Australia) into a slurry and were kept at −20 °C for further analysis.

### 3.3. Extraction of Phenolic Compounds

The extraction was carried out by homogenizing (Ultra-Turrax T25 Homogenizer, IKA, Staufen, Germany) the samples with 70% (*v*/*v*) ethanol for 30 s, followed by incubation (ZWYR-240 incubator shaker, Labwit, Ashwood, VIC, Australia) with 1× *g* Force (RCF), at 4 °C for 12 h. After 12 h, the homogenate was centrifuged (ROTINA 380R, Tuttlingen, Baden-Württemberg, Germany) at 4 °C with 2250× *g* Force (RCF) for 15 min, and the supernatant was collected and diluted with ethanol at appropriate ratios for the various antioxidant analyses [78].

### 3.4. Phenolic Compounds Estimation and Antioxidant Assays

TPC, TFC, and TTC assays were performed to estimate the phenolic contents of the samples, while the antioxidant potentials were determined through DPPH, ABTS, FRAP, and TAC assays according to our previously published protocol [79]. The data were measured using a Multiskan^®^ Go microplate photometer (Thermo Fisher Scientific, Waltham, MA, USA). All assays were run in triplicate, using the standard curves with *R^2^* > 0.995.

#### 3.4.1. Determination of Total Phenolic Content (TPC)

The TPC was determined by modifying the method of Severo and Tiecher [80]. Aliquots of 25 µL of extracts were added into 25 µL of 25% (*v*/*v*) FC reagent + 200 µL Milli-Q water in triplicate in 96-well plates (Costar, Corning, NY, USA). After a 5 min incubation at room temperature, 25 µL of 10% (*w*/*w*) sodium carbonate was added into the reaction mixture and kept in the dark for 1 h at room temperature, followed by absorbance measurements at 764 nm in a plate reader. The absorbance was converted to total polyphenol content based on the calibration curve prepared using the gallic acid standard with concentrations ranging from 0 to 200 µg/mL. The TPC was expressed as mg of gallic acid equivalents (GAE) per gram of the sample (mg GAE/g of the sample), based on fresh weight.

#### 3.4.2. Determination of Total Flavonoids Content (TFC)

The TFC of samples was evaluated by modifying the aluminum chloride method of Ma and Dunshea [78]. Briefly, equal volumes of samples (80 µL) and 2% (*w*/*v*) aluminum chloride ethanolic solution were mixed, followed by the addition of 120 µL of 50 mg/mL sodium acetate. The reaction mixture was incubated at room temperature in the dark for 2.5 h, after which the absorbance was checked at 440 nm. The values of TFC, expressed in quercetin equivalent (µg QE/g _F.W_), were calculated using the standard curve of quercetin (0 to 50 µg/mL).

#### 3.4.3. Determination of Total Tannins Content (TTC)

In the modified method of Zou and Dong [81], an aliquot of 25 µL of sample was added into 150 µL of 4% (*w*/*v*) methanolic vanillin solution, followed by the addition of 25 µL of 32% (*v*/*v*) sulfuric acid in methanol. The reaction mixture was incubated at 25 °C for 15 min, and the absorbance was measured at 500 nm. The absorbance was converted to concentration of tannins with the unit of mg of catechin (0 to 1000 µg/mL) equivalent per gram of sample (mg CE/g _F.W_).

#### 3.4.4. 2,2′-Diphenyl-1-Picrylhydrazyl (DPPH) Antioxidant Assay

Using the modified method of Alvarez-Jubete and Wijngaard [82], the methanolic DPPH (260 µL of 0.1 mM of DPPH) solution was added into 40 µL of sample, followed by incubation for 30 min at room temperature and absorbance measurement at 517 nm. The scavenging activity against DPPH free radicals is expressed as units of ascorbic acid (0 to 50 µg/mL) equivalent (mg AAE/g _F.W_)

#### 3.4.5. Ferric Reducing Antioxidant Power (FRAP) Assay

This assay is based on the principle of reducing the Fe^3+^ in the Fe^3+^-TPTZ complex (ferric-2,4,6-tripyridyl-s-Triazine) into Fe^2+^-TPTZ. The ferric reducing power of samples was estimated according to Chen and Feng [83] with some modifications. The FRAP dye was prepared by mixing sodium acetate solution (300 mM), TPTZ (2,4,6-tripyridyl-s-triazine) solution (10 mM), and Fe[III] solution (20 mM) in a 10:1:1 ratio. A 20 µL aliquot of extract or standard was added to 280 µL of prepared FRAP dye solution in a 96-well plate. Following incubation at 37 °C for 10 min, the absorbance was checked at 593 nm. The results were expressed as mg of ascorbic acid (0 to 150 µg/mL) equivalent per g of fresh sample weight (mg AAE/g _F.W_).

#### 3.4.6. 2′-Azino-Bis-(3-Ethylbenzo-Thiazoline-6-Sulfonic Acid) (ABTS^+^) Radical Scavenging Assay

Following the modified method of Severo and Tiecher [80], free radical ABTS cations were produced by mixing 7 mM ABTS and 140 mM potassium persulfate (625:11 *v*/*v*), then incubating in the dark for 16 h. Further dilution of the stock solution with ethanol was done until an absorbance range of 0.70 ± 0.02 at 734 nm was obtained. Next, this ABTS cation solution (290 µL) was mixed with 10 µL of sample, followed by incubation at room temperature for 6 min and absorbance measurement at 734 nm. The results are expressed as mg of ascorbic acid equivalent per gram of sample (mg AAE/g _F.W_).

#### 3.4.7. Total Antioxidant Capacity (TAC)

TAC of the sample was determined using the phosphomolybdate method [84] with some modifications. The phosphomolybdate reagents used were a combination of 0.004 M ammonium molybdate, 0.028 M sodium phosphate, and 0.6 M sulfuric acid. An aliquoted 40 µL of sample and 260 µL of prepared antioxidant dye was mixed, followed by incubation at 90 °C for 90 min. The mixture was cooled at room temperature for 10 min and absorbance was noted at 695 nm. The results were expressed as mg of ascorbic acid (0 to 200 µg/mL) equivalent per g of fresh sample weight (mg AAE/g _F.W_).

### 3.5. LC-ESI-QTOF-MS/MS Characterization of Phenolic Compounds

The LC-ESI-QTOF-MS/MS analysis was carried out by modifying a previously reported method [78]. HPLC (Agilent 1200 series, Agilent Technologies, Santa Clara, CA, USA) equipped with Accurate-Mass Q-TOF-LC-MS/MS (Agilent 6520 I, Agilent Technologies, Santa Clara, CA, USA) was used for the identification of polyphenolic compounds. The separation of analytes was carried out using a reverse-phase column (Synergi Hydro-RP 80A LC) with an internal diameter of 250 mm × 4.6 mm and particle size of 4 µm (Phenomenex, Lane Cove, NSW, Australia). The column was protected by a Phenomenex C18 ODS guard column with an internal diameter of 4.0 × 2.0 mm. Mobile phase A was an acetic acid/water solution (2:98 *v*/*v*) and mobile phase B was acetonitrile/water/acetic acid (50:0.5:49.5, *v*/*v*/*v*). In total, 6 µL of the sample was injected. The separation was performed using a gradient program with a flow rate of 0.8 mL/min over an 85 min run, where the ratio of the mobile phase B changed from 10% to 25% in 20 min, from 25% to 35% in 10 min, from 35% to 40% in 10 min, from 40% to 55% in 30 min, from 55% to 80% in 5 min, and from 80% to 100% in 2 min, followed by maintenance for 2 min. The ratio of mobile phase B was adjusted to 10% from 100% in 3 min, after the whole separation process, and kept isocratic for 3 min. The samples and column were maintained at 10 °C and room temperature, respectively. The pressure of nitrogen gas condition was set at 45 psi at 300 °C with a flow rate of 5 L/min, while the sheath gas parameter was set with a flow rate of 11 L/min at 250 °C. The capillary and nozzle voltage were set at 3.5 kV and 500 V, respectively. The complete mass scan was in the range of *m*/*z* 50–1300, and MS/MS analyses were carried out in automatic mode with varying collision energy (10, 15, and 30 eV) for fragmentation. The control of the process, data collection, and identification of phenolic compounds was performed using the MassHunter workstation software (Qualitative Analysis, version B.03.01, Agilent, Santa Clara, CA, USA). The compounds tentatively identified by LC-ESI-QTOF-MS/MS with more than 80 library identification scores were further selected for characterization and *m*/*z* verification.

### 3.6. HPLC-PDA Analysis

The samples’ targeted phenolic compounds were quantified using an Agilent 1200 series HPLC (Agilent Technologies, Santa Clara, CA, USA) equipped with a photodiode array (PDA) detector. The same column and conditions were maintained as described above in LC-ESI-QTOF-MS/MS, except for the sample injection volume (20 µL). The compositions of extracts were detected under *λ* 280 nm, 320 nm, and 370 nm by the PDA detector. The individual polyphenols were quantified based on linear regression of external standards, plotting peak area against concentration. Data acquisition and analysis were performed using the Agilent LC-ESI-QTOF-MS/MS MassHunter workstation software (Qualitative Analysis, version B.03.01, Agilent, Santa Clara, CA, USA).

### 3.7. Statistical Analysis

All analyses were performed in triplicate. The values are expressed as mean ± standard deviation (SD). A one-way analysis of variance (ANOVA) with Tukey’s test was carried out using the Minitab^®^ 18 Statistical software (Minitab Inc., State College, PA, USA) for comparisons of antioxidant activities and polyphenol contents between samples. A *p*-value less than 0.05 was considered to denote statistical significance.

## 4. Conclusions

In conclusion, the selected fruits were found to have considerable phenolic contents and antioxidative activities. The TPC, TFC, DPPH, FRAP, ABTS, and TAC values were significantly higher in plums, followed by peaches and pears. It was found that peaches had higher TPC and TFC values than pears, but lower antioxidant performance in DPPH and FRAP assays, which was probably caused by other bioactive compounds in pears, such as vitamin C. As for the identification and characterization of the phenolic compounds in the three selected fruits conducted through LC-ESI-QTOF-MS/MS, a total of 51 phenolic compounds were tentatively identified in the current study, which primarily belonged to the class of phenolic acids and flavonoids. Most of the identified compounds were from plums and pears, belonging to major polyphenolic subclasses such as hydroxybenzoic acids, hydroxycinnamic acids, hydroxyphenylacetic acids, hydroxyphenylpentanoic acids, and flavonols. Regarding the quantification of targeted phenolic compounds in the three fruits, phenolic acids (especially chlorogenic, caffeic, and coumaric) made up the greatest proportion of the plums’ phenolic content, whereas peaches contained more flavonoids (catechin). The phenolic composition was significantly varied among plums, peaches, and pears, which could be one of the reasons responsible for their different antioxidant activities, although this needs further investigation.

## Figures and Tables

**Table 1:** Phenolic estimation and antioxidant activity evaluation of peaches, pears, and plums.

Assays	Peach	Pear	Plums
TPC (mg GAE/g of sample _F.W_)	0.43 ± 0.09 ^b^	0.29 ± 0.05 ^c^	0.62 ± 0.01 ^a^
TFC (µg QE/g of sample _F.W_)	0.29 ± 0.04 ^a^	0.18 ± 0.09 ^b^	0.24 ± 0.07 ^a^
TTC (mg CE/g of sample _F.W_)	0.01 ± 0.08 ^c^	0.03 ± 0.07 ^a^	0.02 ± 0.05 ^b^
DPPH (mg AAE/g of sample _F.W_)	0.20 ± 0.07 ^b^	0.23 ± 0.09 ^b^	0.53 ± 0.08 ^a^
ABTS (mg AAE/g of sample _F.W_)	0.27 ± 0.02 ^b^	0.12 ± 0.07 ^c^	0.47 ± 0.01 ^a^
FRAP (mg AAE/g of sample _F.W_)	0.35 ± 0.04 ^b^	0.41 ± 0.04 ^b^	0.56 ± 0.02 ^a^
TAC (mg AAE/g of sample _F.W_)	0.32 ± 0.09 ^b^	0.19 ± 0.02 ^c^	0.41 ± 0.09 ^a^

TPC, total phenolic content; TFC, total flavonoid content; TTC, total tannin contents; DPPH, 2,2′diphenyl-1-picrylhydrazyl; ABTS, 2,2′-azinobis-3-ethylbenzothiazoline-6-sulfonic acid; FRAP, ferric reducing antioxidant potential; and TAC, total antioxidant capacity. All data are the mean ± SD of three replicates. Means followed by different letters (^a,b,c^) within the same row are significantly different (*p* < 0.05) from each other. Data of three fruits are reported on a fresh weight (_F.W_) basis; mg/g sample ± standard deviation.

**Table 2:** Retention time, mass-to-charge ratio, and characteristic precursor and product ions found for polyphenolic compounds identified in peaches, pears, and plums using LC-ESI-QTOF-MS/MS approaches.

No	Proposed Compound	Molecular Formula	Retention Time (min)	Mode of Ionization	Molecular Weight	Theoretical (*m*/*z*)	Observed (*m*/*z*)	MS/MSProduct Ion	Error (ppm)	Samples
**Phenolic Acids** **Hydroxybenzoic acids**										
**1**	Gallic acid 4-*O*-glucoside	C_13_H_16_O_10_	5.039	[M–H]^−^	332.0743	331.0670	331.0672	169, 125	0.60	* Pear, Plum
**2**	Vanillic acid 4-sulphate	C_8_H_8_O_7_S	5.122	[M–H]^−^	247.9991	246.9918	246.9915	167	−1.21	Pear
**3**	Gallic acid	C_7_H_6_O_5_	6.878	[M–H]^−^	170.0215	169.0142	169.0145	125	1.77	Pear
**4**	5-*O*-Galloylquinic acid	C_14_H_16_O_10_	7.143	[M–H]^−^	344.0740	343.0670	343.0688	191	−0.95	Pear
**5**	2-Hydroxybenzoic acid	C_7_H_6_O_3_	7.607	[M–H]^−^	138.0317	137.0244	137.0244	93	0.00	* Pear, Plum
**6**	4-Hydroxybenzoic acid 4-*O*-glucoside	C_13_H_16_O_8_	11.218	[M–H]^−^	300.0845	299.0772	299.0759	255, 137	−4.35	Pear
**7**	Gallic acid 3-*O*-gallate	C_14_H_10_O_9_	17.066	[M–H]^−^	322.0325	321.0252	321.0239	169	−4.05	Pear
**8**	2,3-Dihydroxybenzoic acid	C_7_H_6_O_4_	32.179	[M–H]^−^	154.0266	153.0193	153.0190	109	−1.96	* Plum, Pear
**Hydroxycinnamic acids**										
**9**	Isoferulic acid 3-sulfate	C_10_H_10_O_7_S	5.341	[M–H]^−^	274.015	273.0074	273.0067	193, 178	−2.56	Plum
**10**	Cinnamic acid	C_9_H_8_O_2_	9.366	[M–H]^−^	148.052	147.0451	147.0444	103	−4.76	* Plum, Pear
**11**	Feruloyl tartaric acid	C_14_H_14_O_9_	10.506	[M–H]^−^	326.064	325.0565	325.0567	193, 149	0.62	Pear
**12**	3-Caffeoylquinic acid	C_16_H_18_O_9_	13.144	[M–H]^−^	354.095	353.0878	353.0874	253, 190, 144	−1.13	Plum
**13**	Caffeic acid	C_9_H_8_O_4_	13.144	** [M–H]^−^	180.042	179.0350	179.0341	143,133	−5.03	* Plum, Pear
**14**	Caffeoyl glucose	C_15_H_18_O_9_	14.833	[M–H]^−^	0342.095	341.0878	341.0887	179, 161	2.64	* Plum, Pear
**15**	3-*p*-Coumaroylquinic acid	C_16_H_18_O_8_	18.180	[M–H]^−^	338.100	337.0929	337.0934	265, 173, 162	1.48	Plum
**16**	Rosmarinic acid	C_18_H_16_O_8_	18.180	[M–H]^−^	360.0845	359.0772	359.0752	179	−5.57	* Plum, Pear
**17**	*m*-Coumaric acid	C_9_H_8_O_3_	18.180	[M–H]^−^	164.0473	163.0400	163.0402	119	1.23	Plum
**18**	Ferulic acid	C_10_H_10_O_4_	18.508	[M–H]^−^	194.0579	193.0506	193.0492	178, 149, 134	−7.25	* Pear, Plum
**19**	Ferulic acid 4-*O*-glucoside	C_16_H_20_O_9_	18.508	[M–H]^−^	356.1107	355.1034	355.1010	193, 178, 149, 134	−6.76	* Pear, Plum
**20**	3-Feruloylquinic acid	C_17_H_20_O_9_	20.847	[M–H]^−^	368.1107	367.1034	367.1025	298, 288, 192, 191	−2.45	* Plum, Pear
**21**	*p*-Coumaric acid 4-*O*-glucoside	C_15_H_18_O_8_	20.897	[M–H]^−^	326.1002	325.0929	325.0922	163	−2.15	* Plum, Pear
**22**	Ferulic acid 4-*O*-glucuronide	C_16_H_18_O_10_	22.305	[M–H]^−^	370.0900	369.0827	369.0826	193	−0.27	* Plum, Pear
**23**	Hydroxycaffeic acid	C_9_H_8_O_5_	37.033	[M–H]^−^	196.0372	195.0299	195.0298	151	−0.51	Plum
**24**	Cinnamoyl glucose	C_15_H_18_O_7_	60.985	[M–H]^−^	310.1053	309.0980	309.0965	147, 131, 103	−4.85	Pear
**Hydroxyphenylpropanoic acids**										
**25**	Dihydroferulic acid 4-*O*-glucuronide	C_16_H_20_O_10_	5.689	[M–H]^−^	372.1056	371.0983	371.0986	195	1.35	Plum
**26**	Dihydrocaffeic acid 3-*O*-glucuronide	C_15_H_18_O_10_	8.468	[M–H]^−^	358.0900	357.0827	357.0811	181	−1.96	Pear
**27**	3-Hydroxy-3-(3-hydroxyphenyl) propionic acid	C_9_H_10_O_4_	14.730	[M–H]^−^	182.0579	181.0506	181.0504	163, 135, 119	−1.1	Pear
**Flavonoids**										
**Dihydroflavonols**										
**28**	Dihydroquercetin	C_15_H_12_O_7_	11.732	[M–H]^−^	304.0583	303.0510	303.0501	285, 275, 151	−2.97	Pear
**Dihydrochalcones**										
**29**	3-Hydroxyphloretin 2′-*O*-glucoside	C_21_H_24_O_11_	13.819	[M–H]^−^	452.1319	451.1246	451.1258	289, 273	−2.22	Pear
**30**	Phloridzin	C_21_H_24_O_10_	51.681	[M–H]^−^	436.1369	435.1326	435.1307	273	−4.37	Peach
**Flavanols**										
**31**	Procyanidin dimer B1	C_30_H_26_O_12_	15.562	[M–H]^−^	578.1424	577.1351	577.1348	451	−0.17	* Plum, Pear, Peach
**32**	Procyanidin trimer C1	C_45_H_38_O_18_	19.240	[M–H]^−^	866.2058	865.1985	865.1961	739, 713, 695	0.69	* Plum, Peach
**33**	Cinnamtannin A2	C_60_H_50_O_24_	19.422	[M–H]^−^	1154.2690	1153.2619	1153.2609	739	−0.87	Plum
**34**	(+)-Catechin	C_15_H_14_O_6_	19.684	[M–H]^−^	290.0790	289.0717	289.0706	245, 205, 179	−3.81	* Pear, Plum
**35**	(+)-gallocatechin	C_15_H_14_O_7_	19.733	[M–H]^−^	306.0739	305.0667	305.0648	261, 219	1.9	Pear
**36**	3′-*O*-Methylcatechin	C_16_H_16_O_6_	24.124	[M–H]^−^	304.0947	303.0874	303.0878	271, 163	1.32	Pear
**37**	(+)-Catechin 3-*O*-gallate	C_22_H_18_O_10_	36.333	[M–H]^−^	442.0900	441.0827	441.0805	289, 169, 125	−0.45	Pear
**Flavanones**										
**38**	Hesperetin 3′-*O*-glucuronide	C_22_H_22_O_12_	47.738	[M–H]^−^	478.1111	477.1068	477.1054	301, 175, 113, 85	−2.93	* Peach, Pear
**Flavones**										
**39**	Apigenin 6,8-di-C-glucoside	C_27_H_30_O_15_	43.862	[M–H]^−^	594.1585	593.1542	593.1539	503, 473	−0.51	Peach
**40**	6-Hydroxyluteolin 7-*O*-rhamnoside	C_21_H_20_O_11_	46.658	[M–H]^−^	448.1006	447.0933	447.0935	301	0.45	* Plum, Pear, Peach
**41**	Apigenin 6-C-glucoside	C_21_H_20_O_10_	55.256	[M–H]^−^	432.1056	431.0983	431.0984	413, 341, 311	0.23	Plum
**Flavonols**										
**42**	Isorhamnetin	C_16_H_12_O_7_	20.284	[M–H]^−^	316.0583	315.0510	315.0504	300, 271	−1.90	Plum
**43**	Quercetin 3-*O*-glucosyl-xyloside	C_26_H_28_O_16_	34.730	[M–H]^−^	596.1377	595.1304	595.1290	265, 138, 116	−2.35	Plum
**44**	Myricetin 3-*O*-rhamnoside	C_21_H_20_O_12_	39.945	[M–H]^−^	464.0955	463.0882	463.0847	317	−7.56	* Pear, Plum, Peach
**45**	Kaempferol 3-*O*-(2″-rhamnosyl-galactoside) 7-*O*-rhamnoside	C_33_H_40_O_19_	42.036	[M–H]^−^	740.2164	739.2091	739.2106	593, 447, 285	2.03	Plum
**46**	Quercetin 3-*O*-arabinoside	C_20_H_18_O_11_	45.598	[M–H]^−^	434.0849	433.0776	433.0780	301	0.92	* Plum, Pear
**Isoflavonoids**										
**47**	6″-*O*-Acetyldaidzin	C_23_H_22_O_10_	4.413	[M–H]^−^	458.1213	457.1140	457.1125	221	−3.28	Plum
**48**	Violanone	C_17_H_16_O_6_	20.267	[M–H]^−^	316.0947	315.0874	315.0868	300, 285, 135	−1.9	Plum
**49**	Glycitin	C_22_H_22_O_10_	30.071	[M–H]^−^	446.1213	447.1286	445.1150	285	2.25	Pear
**Other polyphenols**										
**Hydroxybenzaldehydes**										
**50**	4-Hydroxybenzaldehyde	C_7_H_6_O_2_	44.769	** [M–H]^−^	122.0368	121.0295	121.0298	77	2.48	* Plum, Pear
**Hydroxycoumarins**										
**51**	Coumarin	C_9_H_6_O_2_	20.913	** [M+H]^+^	146.0368	147.0441	147.0448	103, 91	1.38	Plum

RT is short for retention time. Order of product ions listed from higher to lower intensity. * Indicates a compound detected in more than one fruit, ** indicates a compound found in both positive [M+H]^+^ and negative [M–H]^−^ modes.

**Table 3:** Major polyphenol concentrations (mg/100 g of _F.W_) in the flesh of studied fruits.

No.	Compounds Name	RT	Standard Curve	* Peach	* Pear	* Plum
**Phenolic Acids**						
**1**	Gallic acid	6.836	2531.9x + 12238	0.02 ± 0.47 ^b^	4.70 ± 0.98 ^a^	-
**2**	Protocatechuic acid	12.569	1824x − 16182	-	2.10 ± 1.52 ^a^	1.70 ± 2.13 ^ab^
**3**	Caftaric acid	13.774	3500.2x − 43822	0.01 ± 0.91 ^b^	0.30 ± 3.20 ^b^	4.50 ± 3.74 ^a^
**4**	*p*-hydroxybenzoic acid	19.704	1387.5x + 5575.1	3.87 ± 0.94 ^a^	0.90 ± 0.37 ^b^	4.30 ± 1.78 ^a^
**5**	Sinapic acid	38.745	46102x + 724718	3.87 ± 1.78 ^a^	0.93 ± 1.59 ^b^	-
**6**	Ferulic acid	39.823	65160x + 2000000	0.21 ± 3.67 ^b^	0.73 ± 1.20 ^b^	3.44 ± 4.78 ^a^
**7**	Chlorogenic acid	20.579	3043.6x + 4706.3	7.26 ± 3.41 ^ab^	5.26 ± 2.47 ^b^	11.86 ± 3.24 ^a^
**8**	Caffeic acid	25.001	5622.4x + 23944	-	0.09 ± 2.74 ^b^	3.24 ± 0.08 ^a^
**9**	Syringic acid	26.326	2900.6x + 65091	2.23 ± 4.15 ^a^	-	0.03 ± 2.13 ^b^
**10**	Coumaric acid	34.455	6418.4x + 60121	-	-	0.02 ± 1.96 ^a^
**Total phenolic acids**				17.47±15.33 ^b^	15.01±14.07 ^b^	29.09±19.84 ^b^
**Flavonoids**						
**1**	Polydatin	34.966	45035x + 80265	2.45 ± 9.14 ^ab^	3.17 ± 0.15 ^a^	0.89 ± 1.74 ^b^
**2**	Epicatechin gallate	38.015	22958x − 26657	0.75 ± 1.32 ^b^	2.13 ± 0.41 ^b^	3.12 ± 1.96 ^a^
**3**	Catechin	20.240	779.41x + 2373.3	7.31 ± 4.02 ^a^	1.92 ± 3.67^b^	0.30 ± 3.67 ^b^
**4**	Epicatechin	26.739	680.52x + 14866	0.07 ± 3.17 ^b^	1.73 ± 1.29 ^a^	0.49 ± 2.13 ^b^
**5**	q-3-*O*-galactoside	40.659	23472x + 185001	3.21± 1.23 ^a^	0.98 ± 5.21 ^b^	0.06 ± 0.04 ^b^
**6**	Q-3-*O*-rhamnoside	45.172	16282x + 40330	2.12 ± 3.17 ^a^	2.32 ± 1.47 ^a^	-
**7**	kaempferol-3-*O*-glucoside	47.111	22405x − 33766	3.85± 0.04 ^a^	0.03 ± 2.15 ^b^	1.28 ± 6.47 ^b^
**8**	Resveratrol	58.685	7338.8x + 50349	0.01 ± 3.14 ^b^	-	0.09 ± 1.27 ^a^
**9**	Quercetin	70.098	2585.7x − 29267	0.08 ± 7.15 ^b^	-	4.03 ± 3.19 ^a^
**10**	Kaempferol	80.347	4425.8x − 110841	3.03 ± 2.15 ^a^	1.54 ± 3.57 ^b^	2.96 ± 4.12 ^a^
**Total flavonoids**				22.88 ± 34.53 ^a^	13.82 ± 17.92 ^b^	13.22 ± 24.59 ^b^

All values are expressed as mean mg/100 g of sample on fresh basis ± standard deviation (n = 3). Letters indicate the significant difference (*p* < 0.05) in a row using ANOVA and Tukey’s test. * Indicates the quantity of measured polyphenols in respective fruits.

## Data Availability

The data presented in this study are available within the article and Supplementary Materials.

## Supplementary Materials

The following can be downloaded at: https://www.mdpi.com/article/10.3390/metabo12030271/s1. Figure S1: LC-ESI-QTOF-MS/MS basic peak chromatograph (BPC) for the characterization of phenolic compounds of selected fruits from Rosaceae family. Figure S2. Extracted ion chromatogram and their mass spectra in selected fruits from Rosaceae family.
