# Supplementary material for: A Comparative and Comprehensive Characterization of Polyphenols of Selected Fruits from the Rosaceae Family
Source: Metabolites. 2022 Mar 21;12(3):271. doi: 10.3390/metabo12030271 (PMC8950050; doi:10.3390/metabo12030271)
Supplement: Supplementary file 1 [file metabolites-12-00271-s001.zip › metabolites-1627018-supplementary.pdf]

Supplementary data

A Comparative and Comprehensive Characterization of Polyphenols of Selected Fruits from Rosaceae Family

Ahsan Hameed [1,2], Ziyao Liu [2], Hanjing Wu [2], Biming Zhong [2], Michal Ciborowski [1], and Hafiz Ansar Rasul Suleria[2]*

[1] Clinical Research Center, Medical University of Bialystok, Jana Kilińskiego Street 1, 15-089 Bialystok, Poland; ahsanhameed@outlook.com (A.H.); michal.ciborowski@umb.edu.pl (M.C.)

[2] School of Agriculture and Food, Faculty of Veterinary and Agricultural Sciences, The University of Melbourne, Parkville, VIC. 3010, Australia; ziyao.liu@uqconnect.edu.au (Z.L.); hanjingw@student.unimelb.edu.au (H.W.); bimingz@student.unimelb.edu.au (B.Z.); hafiz.suleria@unimelb.edu.au (H.A.R.S.)

* Correspondence: email: hafiz.suleria@unimelb.edu.au; (H.A.R.S). Tel.: +61 3 834 44984

Abstract: The present research presents a comprehensive characterization of polyphenols from peach, pear, and plum using liquid chromatography coupled with electrospray ionization quadrupole-time-of-flight-mass spectrometry (LC-ESI-QTOF-MS/MS) followed by the determination of their antioxidant potential. Plums showed the highest total phenolic content (TPC; 0.62 mg GAE/g), while peaches showed the highest total flavonoid content (TFC; 0.29 mg QE/g), also corresponding to their high scavenging activities (i.e., DPPH, ABTS, FRAP, and TAC). In all three fruit samples, a total of 51 polyphenolic compounds were tentatively identified and characterized mainly from hydroxybenzoic acids, hydroxycinnamic acids, hydroxyphenylpentanoic acids, flavanols, flavonols, and isoflavonoids subclasses. Twenty targeted phenolic compounds were quantified using high-performance liquid chromatography with photodiode array detection (HPLC-PDA). The plum cultivar showed the highest content of phenolic acids (chlorogenic acid, 11.86 mg/100g), whereas peach samples showed the highest concentration of flavonoids (catechin, 7.31 mg/100 g), as compared to pear. Based on these findings, the present research contributes and complements the current characterization data of these fruits presented in the literature and ensures and encourage the utilization of these fruits in different food, feed, and nutraceutical industries.

Keywords: polyphenols; LC-MS; HPLC-PDA; plums; peaches; pears; antioxidant activity; antioxidant components; phenolic acids; flavonoids; flavan-3-ols

a)

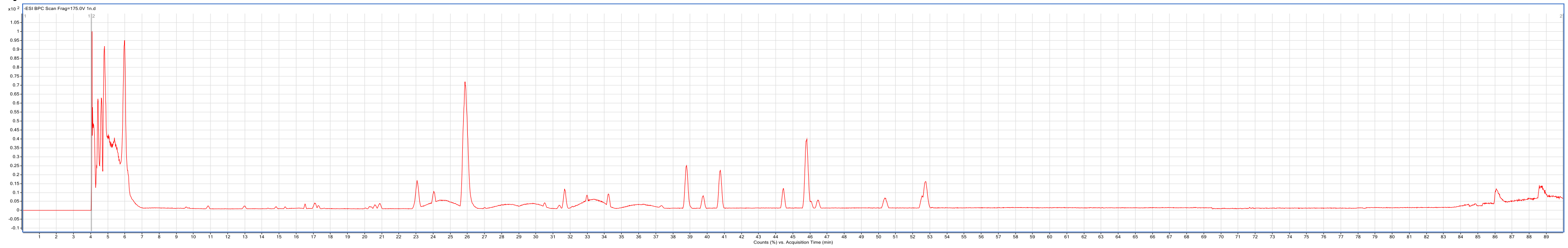
-ESI BPC Scan Frag=175.0V 1n.d
Counts (%) vs. Acquisition Time (min)

b)

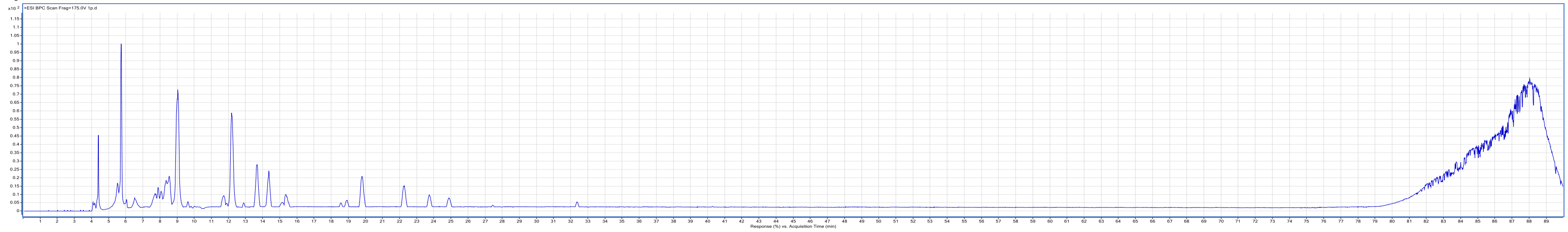
+ESI BPC Scan Frag=175.0V 1p.d
Response (%) vs. Acquisition Time (min)

c)

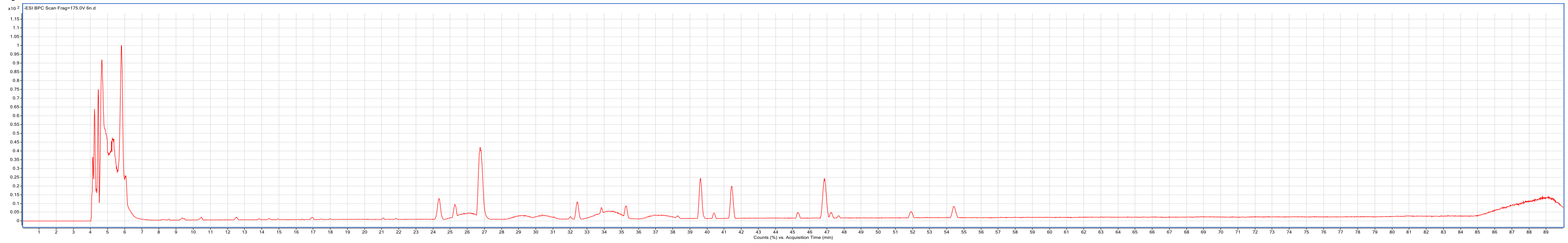
-ESI BPC Scan Frag=175.0V 6n.d
Counts (%) vs. Acquisition Time (min)

d)

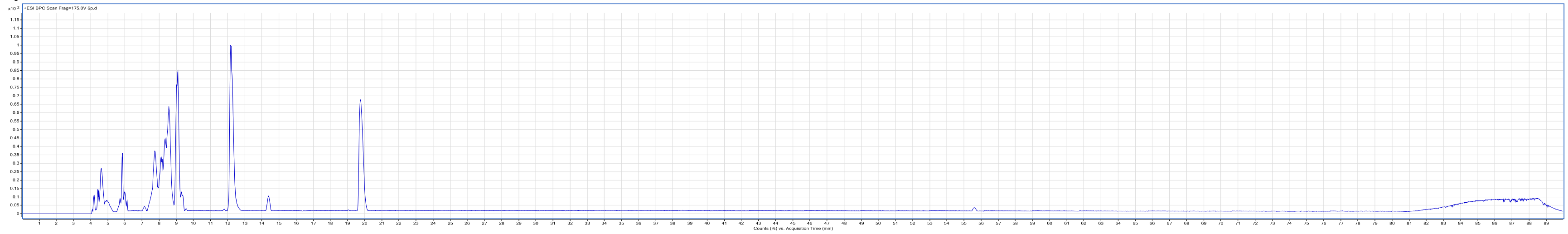
+ESI BPC Scan Frag=175.0V 6p.d
Counts (%) vs. Acquisition Time (min)

e)

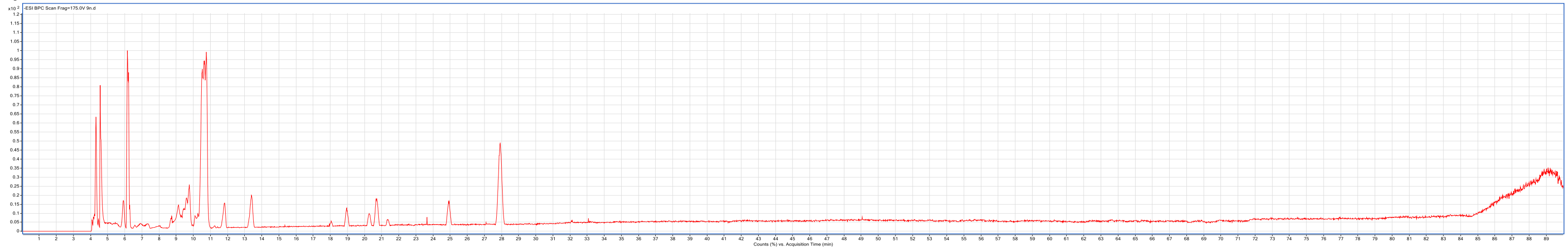

f)

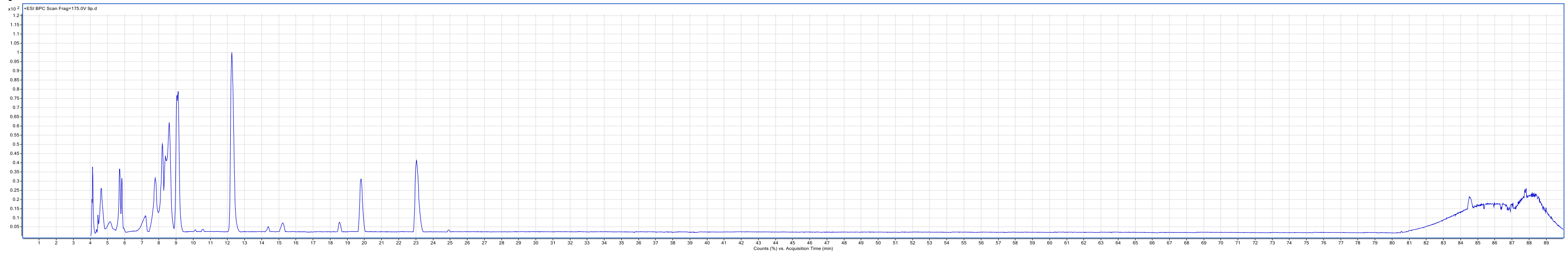

Figure S1. LC-ESI-QTOF-MS/MS basic peak chromatograph (BPC) for characterization of phenolic compounds of selected fruits from *Rosaceae* family; (a) Pear in negative ionization mode; **(b)** Pear in positive ionization mode; **(c)** Peach in negative ionization mode; **(d)** Peach in positive ionization mode; **(e)** Plums in negative ionization mode; **(f)** Plums in positive ionization mode.

a)

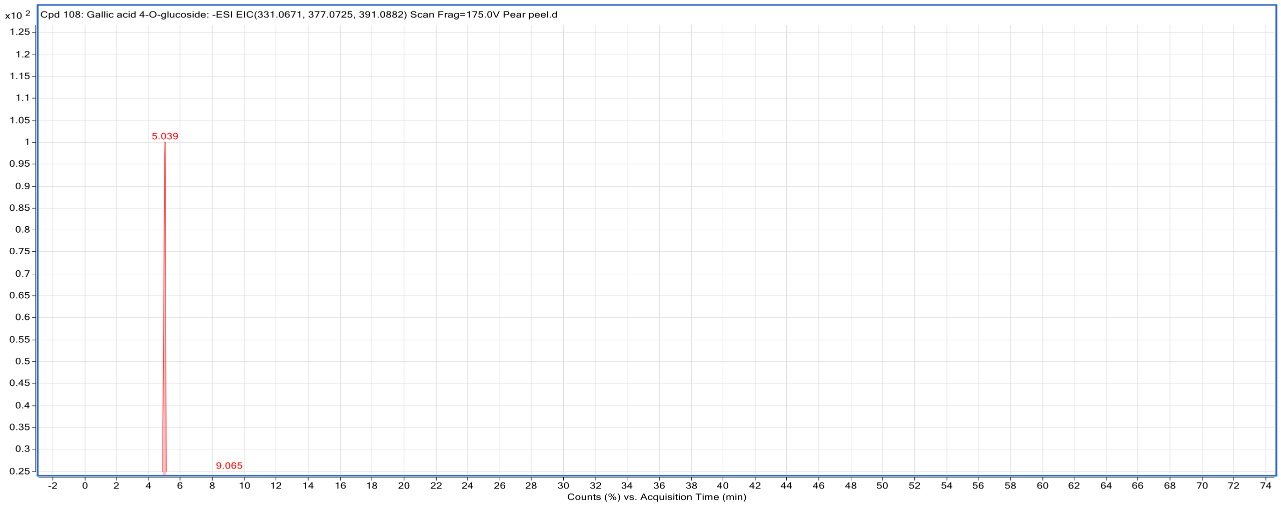

Cpd 108: Gallic acid 4-O-glucoside: -ESI EIC(331.0671, 377.0725, 391.0882) Scan Frag=175.0V Pear peel.d
x10 2
5.039
9.065
Counts (%) vs. Acquisition Time (min)

b)

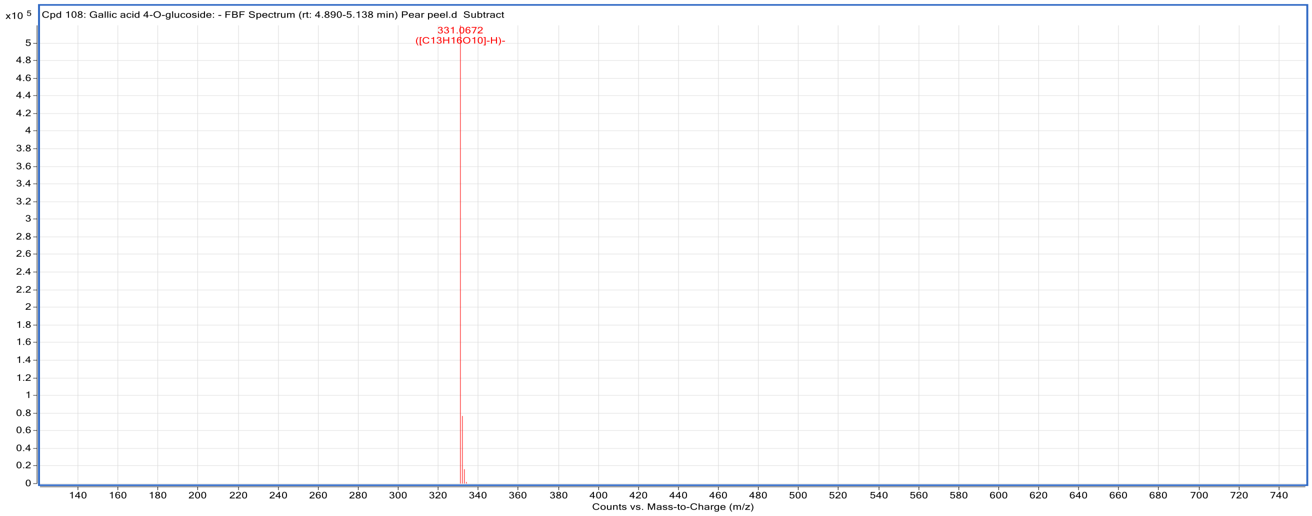

Cpd 108: Gallic acid 4-O-glucoside: - FBF Spectrum (rt: 4.890-5.138 min) Pear peel.d Subtract
x10 5
331.0672
([C13H16O10]-H)-
Counts vs. Mass-to-Charge (m/z)

c)

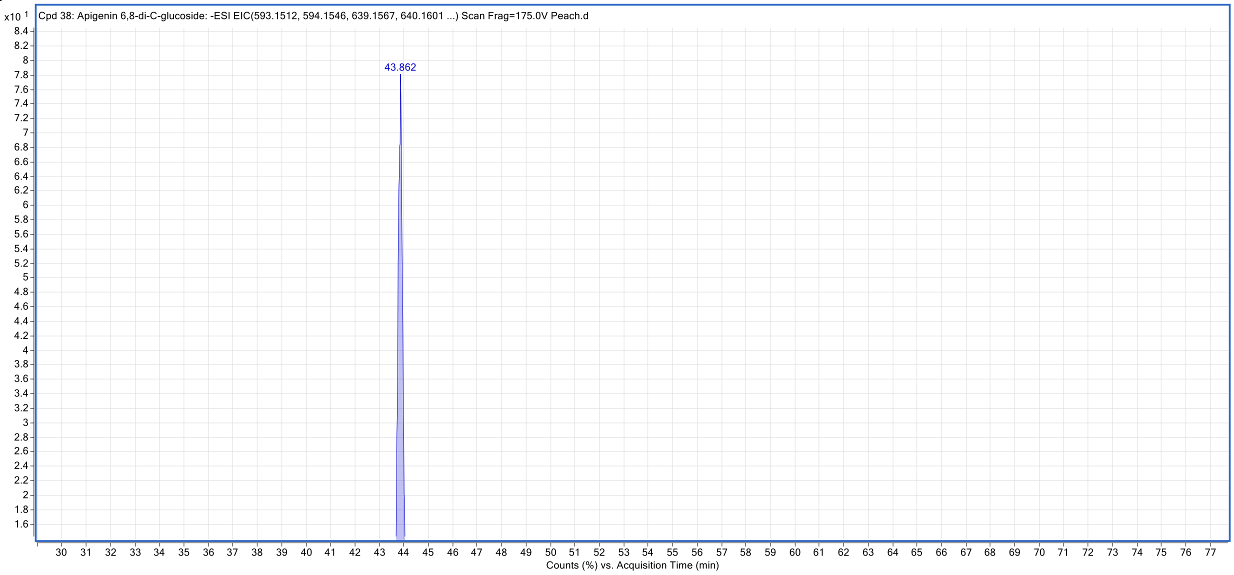
Cpd 38: Apigenin 6,8-di-C-glucoside: -ESI EIC(593.1512, 594.1546, 639.1567, 640.1601 ...) Scan Frag=175.0V Peach.d
x10 1
43.862
Counts (%) vs. Acquisition Time (min)

d)

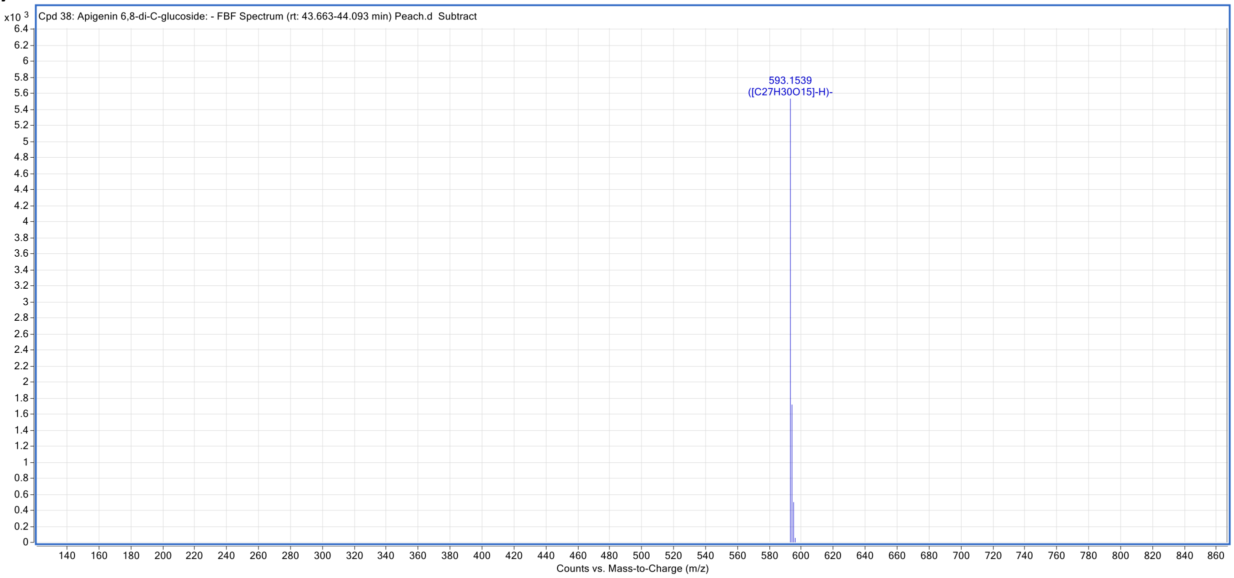
Cpd 38: Apigenin 6,8-di-C-glucoside: - FBF Spectrum (rt: 43.663-44.093 min) Peach.d Subtract
x10 3
593.1539
([C27H30O15]-H)-
Counts vs. Mass-to-Charge (m/z)

e)

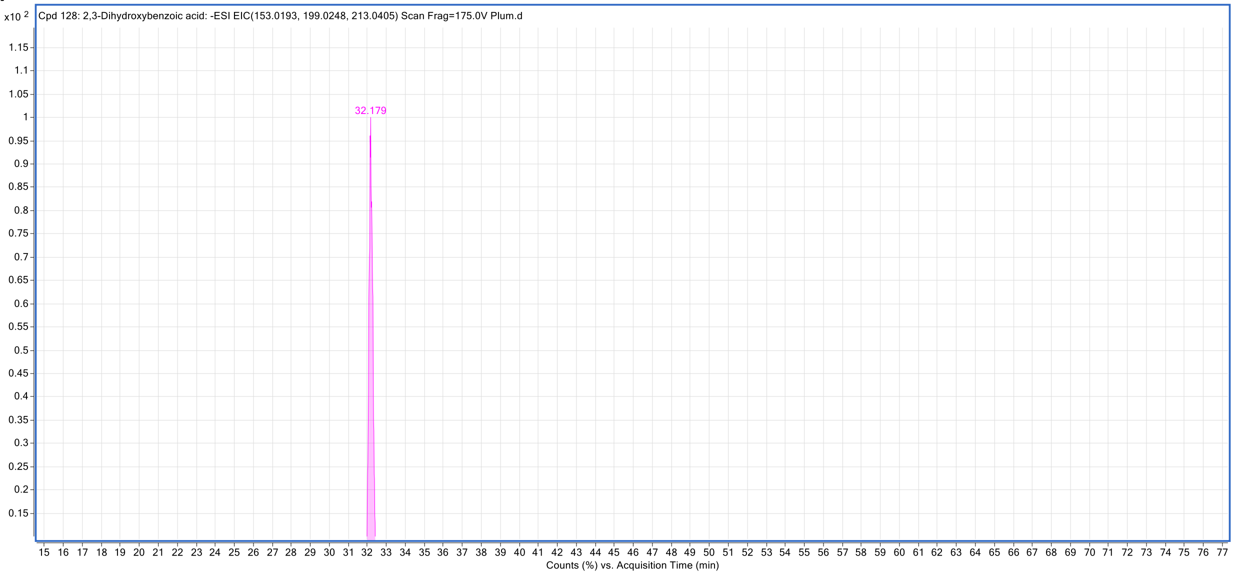

f)

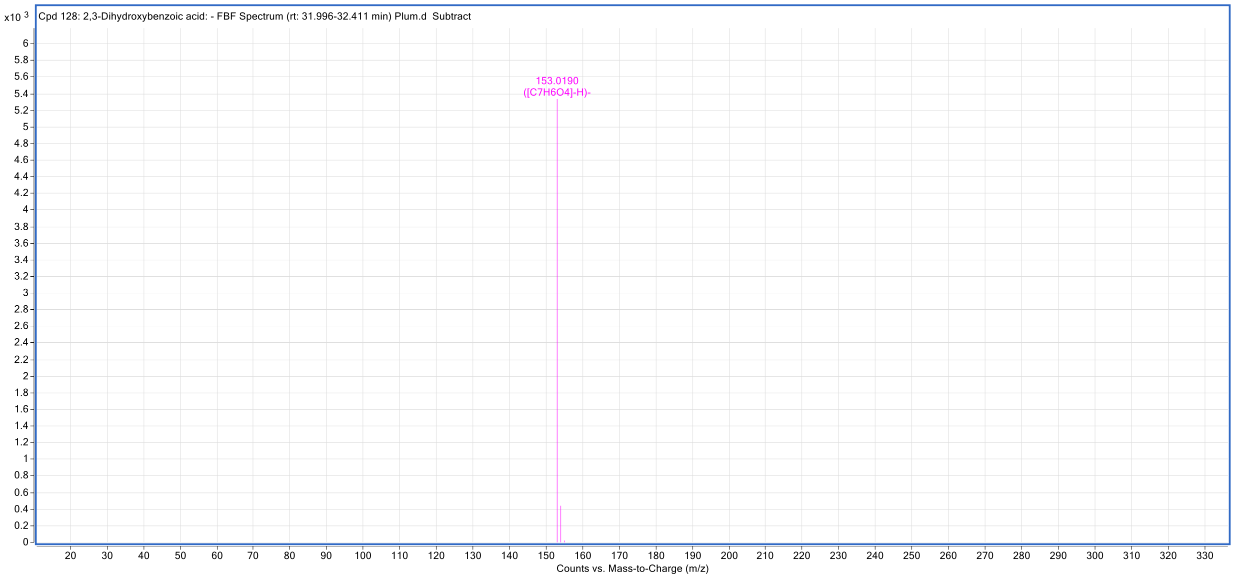

Figure S2. Extracted ion chromatogram and their mass spectrum in selected fruits from Rosaceae family. (a) A chromatograph of Gallic acid 4-*O*-glucoside (Compound **1**, Table 2), Retention time (RT = 5.039 min) in the negative mode of ionization (ESI^-/$[M-H]^-$) identified and characterized in pear sample; **(b)** Mass spectra of Gallic acid 4-*O*-glucoside showing an observed *m/z* 331.0672 in pear sample; **(c)** A chromatograph of Apigenin 6,8-di-C-glucoside (Compound **39**, Table 2), Retention time (RT = 43.862 min) in the negative mode of ionization (ESI^-/$[M-H]^-$) identified and characterized in peach sample; **(d)** Mass spectra of Apigenin 6,8-di-C-glucoside showing an observed *m/z* 593.1539 in peach sample; **(e)** A chromatograph of 2,3-Dihydroxybenzoic acid (Compound **8**, Table 2), Retention time (RT = 32.179 min) in the negative mode of ionization (ESI^-/$[M-H]^-$) identified and characterized in plum sample; **(f)** Mass spectra of 2,3-Dihydroxybenzoic acid showing an observed *m/z* 153.0190 in plum sample.
